# Disclosure of the differences of *Mesorhizobium loti* under the free-living and symbiotic conditions by comparative proteome analysis without bacteroid isolation

**DOI:** 10.1186/1471-2180-13-180

**Published:** 2013-07-31

**Authors:** Yohei Tatsukami, Mami Nambu, Hironobu Morisaka, Kouichi Kuroda, Mitsuyoshi Ueda

**Affiliations:** 1 Division of Applied Life Sciences, Graduate School of Agriculture, Kyoto University, Sakyo-ku, Kyoto 606-8502, Japan; 2 Kyoto Industrial Science and Technology Innovation Center, Shimogyo-ku, Kyoto 600-8813, Japan

**Keywords:** *Mesorhizobium loti*, *Lotus japonicus*, Symbiosis, Proteome analysis, Plant-microbe interaction, Monolithic column, Nitrogen fixation, Rhizobase, KEGG

## Abstract

**Background:**

Rhizobia are symbiotic nitrogen-fixing soil bacteria that show a symbiotic relationship with their host legume. Rhizobia have 2 different physiological conditions: a free-living condition in soil, and a symbiotic nitrogen-fixing condition in the nodule. The lifestyle of rhizobia remains largely unknown, although genome and transcriptome analyses have been carried out. To clarify the lifestyle of bacteria, proteome analysis is necessary because the protein profile directly reflects *in vivo* reactions of the organisms. In proteome analysis, high separation performance is required to analyze complex biological samples. Therefore, we used a liquid chromatography-tandem mass spectrometry system, equipped with a long monolithic silica capillary column, which is superior to conventional columns. In this study, we compared the protein profile of *Mesorhizobium loti* MAFF303099 under free-living condition to that of symbiotic conditions by using small amounts of crude extracts.

**Result:**

We identified 1,533 and 847 proteins for *M. loti* under free-living and symbiotic conditions, respectively. Pathway analysis by Kyoto Encyclopedia of Genes and Genomes (KEGG) revealed that many of the enzymes involved in the central carbon metabolic pathway were commonly detected under both conditions. The proteins encoded in the symbiosis island, the transmissible chromosomal region that includes the genes that are highly upregulated under the symbiotic condition, were uniquely detected under the symbiotic condition. The features of the symbiotic condition that have been reported by transcriptome analysis were confirmed at the protein level by proteome analysis. In addition, the genes of the proteins involved in cell surface structure were repressed under the symbiotic nitrogen-fixing condition. Furthermore, farnesyl pyrophosphate (FPP) was found to be biosynthesized only in rhizobia under the symbiotic condition.

**Conclusion:**

The obtained protein profile appeared to reflect the difference in phenotypes under the free-living and symbiotic conditions. In addition, KEGG pathway analysis revealed that the cell surface structure of rhizobia was largely different under each condition, and surprisingly, rhizobia might provided FPP to the host as a source of secondary metabolism. *M. loti* changed its metabolism and cell surface structure in accordance with the surrounding conditions.

## Background

Rhizobia are nitrogen-fixing soil bacteria that show intracellular symbiosis with their host legume. This symbiotic interaction has become a model system to identify and characterize the attractive mechanism employed by invasive bacteria during chronic host interactions [1]. This symbiosis begins with the secretion of flavonoids by the legume. Subsequently, *nod* genes of rhizobia are activated, and Nod factors (i.e. lipopolysaccharides; LPS) are secreted by rhizobia as signals [2]. After signal exchange between host and symbiont, rhizobia infect the host legume, escaping the vegetative defense responses. The host then produces nodules to maintain symbionts and endocytically incorporates rhizobia into the nodules [3]. In a legume nodule, the host provides C4 dicarboxylates to symbiotic rhizobia as the carbon source; rhizobia fix atmospheric nitrogen and provide ammonia to the host as a nitrogen source in return [4]. Thus, the host plants are able to overcome their nitrogen deficiency.

*Lotus japonicus* and *Mesorhizobium loti* are model organisms of legume-rhizobia symbiosis. The entire genome structures of *L. japonicus* MG-20 and *M. loti* MAFF303099 have been reported previously [5,6], and the database is maintained by the Kazusa DNA Research Institute (Rhizobase; http://genome.microbedb.jp/rhizobase). Transcriptome analysis of *M. loti* by DNA microarray revealed that most of the transposase genes and *nif*, *fix*, *fdx*, and *rpoN* on the symbiosis island were highly upregulated under the symbiotic condition, while genes for cell wall synthesis, cell division, DNA replication, and flagella formation were strongly repressed under the symbiotic condition [7]. However, less information is available about *M. loti* than about other genera of rhizobia, such as *Sinorhizobium meliloti, Rhizobium leguminosarum,* and *Bradyrhizobium japonicum*.

In addition to transcriptome analysis, proteome analysis has recently attracted much attention. While genome sequencing has provided a considerable amount of useful information to clarify biological phenomena, proteins, rather than genes, actually function *in vivo*. Thus, it is necessary to analyze the entire set of produced proteins [8]. Proteome analysis of *M. loti* in mid-growth phase has been reported [9], but it has not been performed for the symbiotic phase. Proteome analyses of other rhizobia, such as *B. japonicum*[10-14], and *S. meliloti*[15-20], have been previously reported. They employed 2-dimensional polyacrylamide gel electrophoresis (2D-PAGE)-based analysis combined with matrix-assisted laser desorption and ionization time-of-flight mass spectrometry (MALDI-MS), but time-consuming steps, such as gel spot isolation and individual measurement, are necessary in this method. In addition, previous 2D-PAGE-based analyses have only identified up to 500 proteins [13]. Another report employed liquid chromatography-tandem mass spectrometry (LC-MS/MS)-based technology combined with prefractionation, such as multidimensional chromatography [21] or gel-based separation [22], but these prefractionation steps decreased throughput. Furthermore, all of them included a complicated isolation step of the bacteroid (a symbiotic form of rhizobia) from the nodule, and the step required a large amount of biological samples, such as 1–5 g nodules collected from approximately 40 plants [23]. Detection of small amount of proteins present in complex biological samples remains difficult and requires a combination of prefractionation steps.

To solve the problems, we used a nanoLC-MS/MS system equipped with a long monolithic silica capillary column (200 cm long, 0.1 mm ID). Monolithic silica materials offer high separation efficiency in long column formats because of their high permeability [24], and they have been successfully applied to separate tryptic fragments in highly complex samples with a shallow gradient. As this high-resolution system does not require any additional prefractionation prior to the separation and detection step by LC-MS/MS, this approach can simplify the workflow of shotgun proteomics and minimize the sample amount, as well as total analysis time [25]. Using this system, we have successfully performed proteome analysis of *Candida albicans*[26] and *Clostridium cellulovorans*[27].

Here, we report the first comparative proteome analysis of *M. loti* under the free-living and symbiotic conditions by using our system. Our data should accelerate functional and comprehensive studies focused on molecular mechanisms of *L. japonicus* - *M. loti* symbiosis.

## Results and discussion

### Identification of proteins extracted from free-living and symbiotic *M. loti*

**Figure 1:**
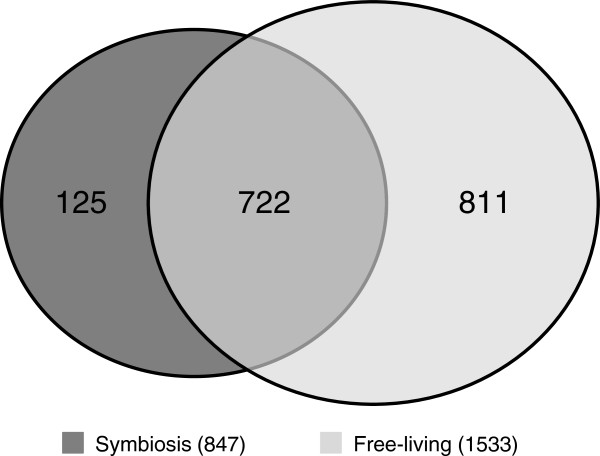
**Venn diagram of proteins identified in *M. loti. *** A total of 1,658 proteins were identified. Although 722 proteins were commonly identified under the free-living and symbiotic conditions, 811 and 125 proteins were uniquely identified under the free-living and symbiotic conditions, respectively.

The tryptic digests were injected to a LC-MS/MS system equipped with a long monolithic silica capillary column; 1,658 proteins were successfully identified by efficient separation (Additional file 1). Specifically, 1,533 proteins were identified under the free-living condition, and 847 proteins were identified by the analytes extracted from nodules without bacteroid isolation and prefractionation (Figure 1). Many proteins encoded in the symbiosis island were also identified. The symbiosis island of *M. loti* MAFF303099 is one of the notable features, which occurs by integration of a horizontally transferred DNA segment, and is located on a 610,975-bp DNA segment of the chromosome at coordinates 4,644,702 to 5,255,766 [5]. A total of 582 protein-encoding genes were located on the symbiosis island. Mapping the identified proteins to the symbiosis island showed that 74 proteins (8.7% of 847 proteins) were produced under the symbiotic condition, whereas only 22 proteins (1.4% of 1,533 proteins) were produced under the free-living condition. From the viewpoint of reproducibility, our data show highly-reproducible result with the strict criteria for protein identification (Additional file 2). As shown in this figure, 87% of proteins were identified from 3 data set under the free-living conditions, although the previous report indicated that protein profile of free-living *M. loti* in stationary phase was not reproducible [9]. And identified proteins under the symbiotic condition also show high-reproducibility because 84% of proteins were identified at all measurements. These results indicated that the protein profile successfully obtained with our system reflected the free-living and the symbiotic conditions.

### KEGG pathway analysis

For further investigation about the lifestyle of rhizobia under each condition, the identified proteins were classified according to the Kyoto Encyclopedia of Genes and Genomes (KEGG; http://www.genome.jp/kegg/), and metabolic pathways were compared under the free-living and symbiotic conditions. The number of classified enzymes in each pathway is shown in Table 1, and the annotated genes in Table 1 are listed in Additional file 3.

**Table 1:** The number of classified enzymes detected by proteome analysis

**Pathway**	**Symbiotic condition**	**Free-living condition**	**Genes**^**a)**^
Central carbon metabolism	49	56	77
Nitrogen fixation	8	2	8
Ubiquinone biosynthesis	6	5	9
Nucleotide sugar metabolism	1	6	13
Peptidoglycan biosynthesis	2	7	15

^a)^The number of genes proposed by KEGG pathway analysis.

#### Central carbon metabolism

**Figure 2:**
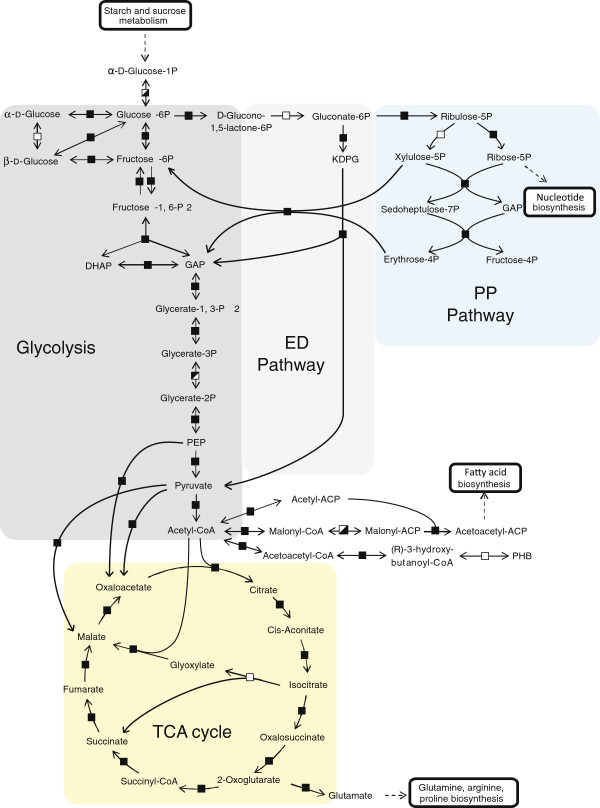
**The map of central carbon metabolic pathways under the free-living and/or symbiotic conditions.** The map of central carbon metabolic pathways involving gluconeogenesis and the Embden-Meyerhof-Parnas (EMP) pathway, the Entner-Doudoroff (ED) pathway, the pentose phosphate (PP) pathway, and the TCA cycle are shown. Symbols represent the following: fully-filled box (■), enzymes that were commonly identified under each condition; boxes filled in the bottom-right corner (◪), enzymes identified only under the free-living condition; boxes filled in the upper-left corner (◩), enzymes that were identified only under the symbiotic condition; open box (□), enzymes not identified in this study but proposed in *M. loti* by KEGG pathway analysis. Abbreviations are as follows: DHAP, dihydroxyacetone phosphate; GAP, glyceraldehyde-3-phosphate; PEP, phosphoenolpyruvate; KDPG, 2-dehydro-3-deoxy-phosphogluconate; ACP, acyl carrier protein; PHB, polyhydroxybutyrate.

Most enzymes classified in carbon metabolism, such as glycolysis, gluconeogenesis, TCA cycle, pentose phosphate (PP), and Entner-Doudoroff (ED) pathways, were commonly identified (Figure 2). It is assumed that the same pathways located in central carbon metabolism remained largely unchanged, irrespective of conditions.

**Figure 3:**
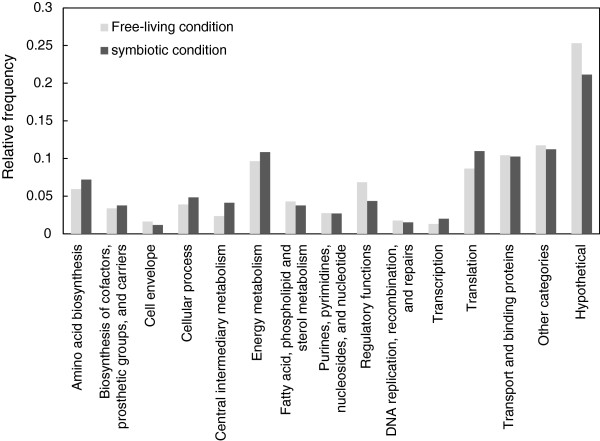
**Functional classification according to Rhizobase.** Relative frequency of genes/proteins belonging to a category is given for 2 data sets: the proteins detected under the free-living condition (1,533) (dark gray) and in the *L. japonicus* nodule (847) (light gray). The relative frequencies were calculated by dividing the number of proteins into each category by the total number of identified proteins.

To investigate the functional distribution, identified proteins under each condition were classified into 15 major functional categories according to Rhizobase (Figure 3). There was no significant difference between the functional profiles under each condition. (Statistical significances were determined using Pearson’s chi-square test, *p* > 0.01). This indicated that the metabolic pathways, which constitute the backbone of life, were commonly used under both conditions.

#### Nitrogen fixation

**Figure 4:**
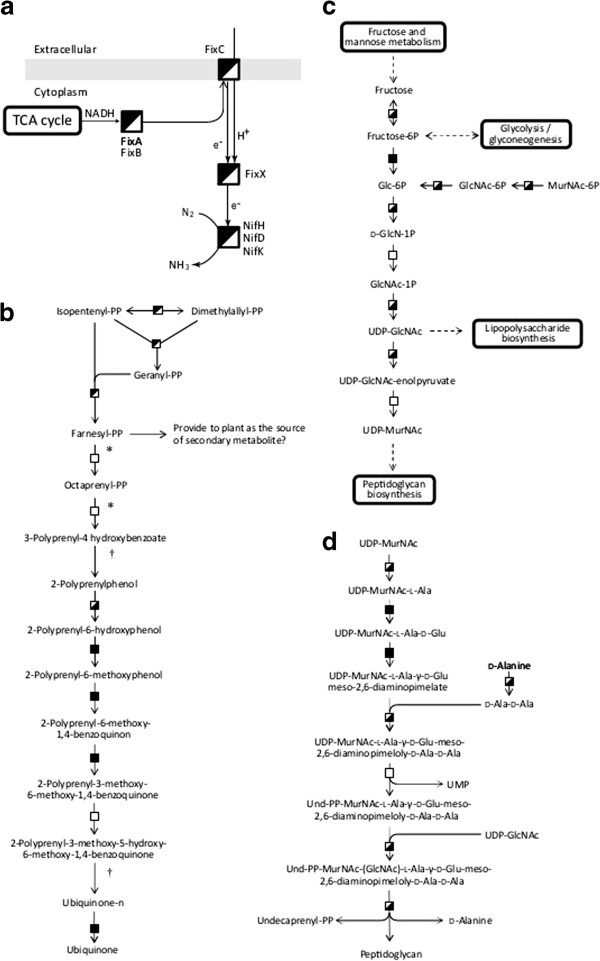
**The map of metabolic pathways under the symbiotic and/or free-living conditions.** The map of metabolic pathways is shown: **(a)** nitrogen fixation, **(b)** ubiquinone biosynthesis, **(c)** amino sugar metabolism, **(d)** peptidoglycan biosynthesis. Box symbols indicate the same things as in Figure 2. Daggers (†) indicate the reactions that have universally existed but have not been proposed in *M. loti* by KEGG pathway analysis. Asterisks indicate the focused enzymes discussed in the “Results and discussion” section. Abbreviations are as follows: GlcN, Glucosamine; GlcNAc, *N*-acetyl-*D*-glucosamine; MurNAc, *N*-acetylmuramic acid.

Nitrogenase complex core subunits (NifH, NifD, NifK) and the electron donor proteins (FixA, FixB, FixC), which transfer electrons to the nitrogenase complex, were detected only under the symbiotic condition (Figure 4a). Fixation of atmospheric nitrogen is a characteristic feature of rhizobia only under the symbiotic condition [7]. The proteins related to nitrogen fixation, such as nitrogenase construction (NifN, NifX, NifS, NifW) [28], electron donation (FixX, FixP), and symbiosis-unique ferredoxins (mlr5869, mlr5930, msl8750), were also found to be unique to the symbiotic condition. In addition, NifA and RpoN, which are known to cooperatively regulate *nif* and *fix* genes, were detected only under the symbiotic condition [29]. The protein profile strongly reflected the phenotype that was predicted by transcriptome analysis [7].

#### Farnesyl diphosphate (FPP) biosynthesis

It is generally known that rhizobia provide ammonia and other amino acids as a nitrogen source to the host [4], while no other compound is known to be provided. However, the obtained protein profile suggested that FPP might be provided from rhizobia to plant root cells. In the quinone biosynthetic pathway, the enzymes necessary to FPP biosynthesis, such as isopentenyl pyrophosphate isomerase (mlr6371) and geranyltransferase (mlr6368), which are located in the rhizobia symbiosis island, were uniquely detected under the symbiotic condition (Figure 4b). These enzymes produce FPP from isopentenyl diphosphate and dimethyl allyl diphosphate. FPP is an intermediate in the mevalonate pathway, which is present in all higher eukaryotes and many bacteria. FPP is used for the biosynthesis of ubiquinone in *M. loti*. However, the enzymes which catalyze the ubiquinone biosynthesis reactions from FPP (shown in asterisks in Figure 4b) were not detected at the protein level. Additionally, the symbiosis island does not include genes encoding octaprenyl-diphosphate synthase (mlr7426) and 4-hydroxybenzoate polyprenyltransferase (mll7442), which are involved in the pathway of ubiquinone biosynthesis. On the other hand, higher plants utilize FPP as the intermediate precursor of many secondary metabolites, such as sesquiterpenes, triterpenes, and sterols [30]. It is reasonable to suppose that FPP is provided to the host legume from rhizobia as a source of secondary metabolites because FPP was synthesized only under the symbiotic condition, as the enzymes that metabolize FPP after its production were not detected in *M. loti* at the protein level. However, the estimation is just based on the obtained protein profile, and further investigation of the migration of FPP will be carried out by using deletion mutants, and by analysis at mRNA and metabolite levels.

#### Nucleotide sugar metabolism and peptidoglycan biosynthesis

On the other hand, the enzymes involved in uridine diphosphate (UDP) sugar metabolism were not produced under the symbiotic condition (Figure 4c), and LPS transporters (mll3197, mll7564, mll7866) were not produced under the symbiotic condition. UDP-*N*-acetylglucosamine (UDP-MurNAc) is the starting material for LPS biosynthesis. LPS is known as one of the “nod factors,” which is secreted by the rhizobial body when it perceives the root through the flavonoid groups secreted from host legume [2]. The secretion of LPS is likely unnecessary under the symbiotic condition (after infection). In addition, UDP-*N*-acetylmuramic acid, the end product of this pathway, is the starting material of peptidoglycan biosynthesis. The enzymes of peptidoglycan biosynthesis were uniquely detected under the free-living condition (Figure 4d).

Under the symbiotic condition, rhizobia are differentiated into a bacteroid, and the peribacteroid membrane (PBM)-enclosed bacteroids are essentially a nitrogen-fixing intracellular organelle, termed the ‘symbiosome.’ In PBM, bacteroids are stationary and become slightly larger than the free-living rhizobia [31]. However, the remarkable structural changes have not been confirmed at the protein level. Proteome data could detect the proteins involved in the structural changes, as well as changes in metabolic pathway; thus, we focused on cell surface structure.

From our data, it was predicted that peptidoglycan was not biosynthesized under the symbiotic condition described above (Figure 4d). Peptidoglycan, which is the main material of bacterial cell wall, plays an important role in the maintenance of structure by providing tolerance to osmotic pressure and mechanical stress, and it is also involved in cell division during growth [32]. The inactivation of the peptidoglycan biosynthetic pathway under the symbiotic condition is supported by the following: (1) the neogenesis of peptidoglycan is unnecessary because fully symbiotic rhizobia cease their cell division, (2) symbiotic rhizobia are able to avoid mechanical stress because of enclosure by PBM and immobility, and (3) the host legume might control the surrounding environment not to impose an osmotic stress on rhizobia. The protein profile indicates that the interruption of peptidoglycan biosynthesis in symbiotic *M. loti* occurs at the protein level, and rhizobia under the symbiotic condition might lose its cell wall.

#### Flagellum and pilus components

We investigated structural proteins, such as flagellum and pilus components. The flagellum is connected to bacterial motility and attachment of rhizobia to developing root hairs, which is one of the first steps of nitrogen-fixing root nodule symbiosis [33]. The pilus is a hair-like appendage found on the surface of many bacteria and is related to the process of bacterial conjugation. Rhizobia have not only conjugative pili but also type IV pili, which generate motile forces called twitching motility, in which the pilus works as a grappling hook to bind to a variety of surfaces [34]. The flagellum component proteins, FlaA (mlr2925, mlr2927), FlgL (mlr2939), FlgK (mlr2938), MotB (mlr3926), and FliN (mll2902), were detected only under the free-living condition. DNA microarray analysis has shown that the gene of flagellar L-ling protein (FlgH; mll2921) is repressed at the mRNA level [7]. Therefore, the obtained protein profile confirmed that under the symbiotic condition, rhizobia repress flagellum genes, and it also indicated that structural proteins of the flagellum are not present under the symbiotic condition. In addition, the pilus assembly proteins, CpaB (mll5595), CpaD (mll5598), and CpaE (mll5600), were also detected only under the free-living condition. Flagella and pili were lost under the symbiotic condition because rhizobia under the symbiotic condition would have no need for conjugation, infection, and motility in PBM. In contrast, rhizobia under the free-living condition require the flagellum and pilus component proteins.

## Conclusion

**Figure 5:**
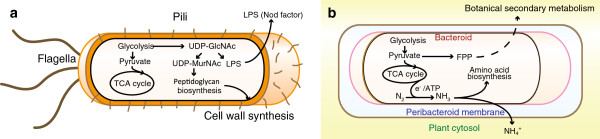
**Schematic representation of the lifestyle under the symbiotic condition compared to the free-living condition.** The illustration shows the changes in the lifestyles of *M. loti*: the lifestyle model under the **(a)** free-living and **(b)** symbiotic conditions. The central carbon metabolic pathway is essential under both conditions. Under the symbiotic condition, nitrogen is fixed by electrons from the TCA cycle or other energy metabolism and is provided to the host legume or used for amino acid biosynthesis. Moreover, the flagellum and pilus are lost, and the cell wall, which is mainly composed of peptidoglycan, may become thin or disappear. In contrast, FPP is synthesized to provide to the host legume. Under the free-living condition, LPS is secreted extracellularly as a nod factor to infect the host legume.

In order to detect the changes in *M. loti* between free-living and symbiotic conditions, we performed proteome analysis of *M. loti*. We used our LC-MS/MS system, equipped with a long monolithic silica capillary column, to successfully identify 1,658 proteins without bacteroid isolation and prefractionation. This analytical system opens up a new horizon for symbiotic proteome analysis from small amounts of unpurified crude biological samples. The protein profile indicated some interesting and unexpected results associated with the cell surface structure and metabolism, in accordance with the external environment of each condition (Figure 5). The data set revealed that *M. loti* under the symbiotic condition simplifies the components of the cell surface, such as flagellum, pilus, and cell wall. In addition, we found that *M. loti* under the symbiotic condition provided not only a nitrogen source but also FPP, which is a source of secondary metabolism. Our data should be helpful in carrying out detailed studies on the change of these 2 conditions of rhizobia.

## Methods

### Strains and growth conditions

*M. loti* MAFF303099 was cultured in tryptone-yeast extract (TY) medium [35] at 28°C. Cells were harvested in the early stationary phase for 72 h. Cells were subjected to sample preparation in the free-living condition.

For the symbiotic condition, *L. japonicus* MG-20 Miyakojima [36] seeds were sterilized, germinated, and inoculated with *M. loti* and grown in MM1 [37] medium at 25°C with a 16-h light/8-h dark cycle. Root nodules from several plants were harvested at 7 weeks post-inoculation. Nodules from 3 independently grown pools of plants were collected and processed in parallel.

Nodules were frozen with liquid nitrogen, homogenized with an ice-cold mortar, and subjected to sample preparation.

### Sample preparation

Collected cells were resuspended with 500 μL of lysis buffer (2% (*w*/*v*) 3-(3-cholamidopropyl)dimethylammonio-1-propanesulfonate, 10 mM dithiothreitol, 1% (*v*/*v*) protease inhibitor cocktail (Sigma-Aldrich, St. Louis, MO, USA), 7 M urea, and 2 M thiourea in 50 mM Tris–HCl (Nacalai tesque, Kyoto, Japan)). The solution was mixed with an equal volume of 0.5-mm glass beads (Tomy Seiko, Tokyo, Japan). The cells were then disrupted mechanically in triplicate by using BeadSmash 12 (Wakenyaku, Kyoto, Japan) at 4°C, 4,000 × *g* for 1 min. The solution was centrifuged at 14,000 × *g* for 10 min, and the supernatant was collected. The supernatant was filtered by 0.45 μm Ultrafree-MC (Millipore, Billerica, MA, USA). The filtered solution was subjected to ultrafiltration using Amicon Ultra YM-10 (Millipore) and buffer-exchanged by 200 mM triethyl ammonium bicarbonate (TEAB; Sigma-Aldrich). The proteins were reduced by adding 10 mM tris-(2-carboxyethyl)phosphine (Thermo Fisher Scientific, Waltham, MA, USA) and incubated at 55°C for 1 h. After the reaction, 20 mM iodoacetamide was added to the solution, and incubated for 30 min. The reactant was mixed with 1 mL of ice-cold acetone and incubated at −20°C for 3 h to precipitate proteins. The precipitated proteins were resuspended with 100 μL of 200 mM TEAB and mixed with 2 μl (1 μg μL^-1^) of sequencing grade modified trypsin (Promega, Madison, WI, USA) at 37°C overnight. The peptide concentration of the tryptic digests was measured using Protein Assay Bicinchoninate Kit (Nacalai tesque). The concentrations of the injected digests were 1.06 ± 0.12 μg μL^-1^ digest for free-living *M. loti* and 4.96 ± 0.90 μg μL^-1^ digest for nodules, respectively. (mean ± SD, N = 3).

### LC-MS/MS analysis

Proteome analyses were performed by a liquid chromatography (UltiMate3000 RSLCnano system (Thermo Fisher Scientific))/mass spectrometry (LTQ Velos mass spectrometer (Thermo Fisher Scientific)) system equipped with a long monolithic silica capillary column (200-cm long, 0.1-mm ID) [24,27]. 10 and 5 μL of tryptic digests were injected for free-living and symbiotic conditions, respectively, and separated by reversed-phase chromatography at a flow rate of 500 nL min^-1^. The gradient was provided by changing the mixing ratio of the 2 eluents: A, 0.1% (*v/v*) formic acid and B, 80% (*v/v*) acetonitrile containing 0.1% (*v/v*) formic acid. The gradient was started with 5% B, increased to 50% B for 600 min, further increased to 95% B to wash the column, then returned to the initial condition, and held for re-equilibration. The separated analytes were detected on a mass spectrometer with a full scan range of 350–1,500 *m/z*. For data-dependent acquisition, the method was set to automatically analyze the top 5 most intense ions observed in the MS scan. An ESI voltage of 2.4 kV was applied directly to the LC buffer end of the chromatography column by using a MicroTee (Upchurch Scientific, Oak Harbor, WA, USA). The ion transfer tube temperature was set to 300°C. Triplicate analyses were done for each sample of 3 biological replicates, and blank runs were inserted between different samples.

### Data analysis

The mass spectrometry data of each sample were used for protein identification using MASCOT (Matrix Science, London, UK), working on Proteome Discoverer (Thermo Fisher Scientific) against the database at Rhizobase containing 7,283 sequences with a peptide tolerance of 1.2 Da, MS/MS tolerance of 0.8 Da, and maximum number of missed cleavages of 2. For trypsin digestion, cysteine carbamidomethylation (+57.021 Da) and methionine oxidation (+15.995 Da) were set as a variable modification. The data were then filtered at a q-value ≤ 0.01 corresponding to 1% false discovery rate on a spectral level. Moreover, proteins identified by at least 2 peptides per protein or identified by a single peptide per protein at any 3 data points were accepted as ‘identified proteins.’

The pathway analysis of identified proteins is performed by using the pathway mapping tool on KEGG (http://www.genome.jp/kegg/). The functional classification of proteins was performed by using Rhizobase at Kazusa DNA Research Institute (http://www.kazusa.or.jp/e/index.html).

## Competing interests

The authors declare that they have no conflict of interest.

## Authors' contributions

YT and MN generated the strains used. YT and HM performed most of the analyses. YT, HM, KK and MU designed the study and drafted the manuscript. All authors read and approved the final manuscript.

## Supplementary Material

Additional file 1 **List of identified proteins under each condition.** a) Average of Mascot score of 3 measurements in protein identification. b) Peptides per protein in protein identification. c) Not detected. Click here for file

Additional file 2 **The Venn diagrams of identified proteins at each measurement (N = 3).** The number of identified proteins were shown in bold, and percentages were indicated between brackets. Click here for file

Additional file 3 **The annotated genes by the KEGG pathway analysis in Table**1. a) Average of Mascot score of 3 measurements in protein identification. b) Peptides per protein in protein identification. c) Not detected. Click here for file

## References

[B1] Gibson KE Kobayashi H Walker GC Molecular determinants of a symbiotic chronic infection Annu Rev Genet 2008 42 413 441 10.1146/annurev.genet.42.110807.091427 18983260 PMC2770587

[B2] Denarie J Debelle F Prome JC Rhizobium lipo-chitooligosaccharide nodulation factors: Signaling molecules mediating recognition and morphogenesis Annu Rev Biochem 1996 65 503 535 10.1146/annurev.bi.65.070196.002443 8811188

[B3] Oldroyd GE Downie JA Coordinating nodule morphogenesis with rhizobial infection in legumes Annu Rev Plant Biol 2008 59 519 546 10.1146/annurev.arplant.59.032607.092839 18444906

[B4] Prell J Poole P Metabolic changes of rhizobia in legume nodules Trends Microbiol 2006 14 161 168 10.1016/j.tim.2006.02.005 16520035

[B5] Kaneko T Nakamura Y Sato S Asamizu E Kato T Sasamoto S Watanabe A Idesawa K Ishikawa A Kawashima K Kimura T Kishida Y Kiyokawa C Kohara M Matsumoto M Matsuno A Mochizuki Y Nakayama S Nakazaki N Shimpo S Sugimoto M Takeuchi C Yamada M Tabata S Complete genome structure of the nitrogen-fixing symbiotic bacterium *Mesorhizobium loti* DNA Res 2000 7 381 406 10.1093/dnares/7.6.381 11214974

[B6] Sato S Nakamura Y Kaneko T Asamizu E Kato T Nakao M Sasamoto S Watanabe A Ono A Kawashima K Fujishiro T Katoh M Kohara M Kishida Y Minami C Nakayama S Nakazaki N Shimizu Y Shimpo S Takahashi C Wada T Yamada M Ohmido N Hayashi M Fukui K Baba T Nakamichi T Mori H Tabata S Genome structure of the legume, *Lotus japonicus* DNA Res 2008 15 227 239 10.1093/dnares/dsn008 18511435 PMC2575887

[B7] Uchiumi T Ohwada T Itakura M Mitsui H Nukui N Dawadi P Kaneko T Tabata S Yokoyama T Tejima K Saeki K Omori H Hayashi M Maekawa T Sriprang R Murooka Y Tajima S Simomura K Nomura M Suzuki A Shimoda Y Sioya K Abe M Minamisawa K Expression islands clustered on the symbiosis island of the *Mesorhizobium loti* genome J Bacteriol 2004 186 2439 2448 10.1128/JB.186.8.2439-2448.2004 15060047 PMC412173

[B8] Tyers M Mann M From genomics to proteomics Nature 2003 422 193 197 10.1038/nature01510 12634792

[B9] Kajiwara H Kaneko T Ishizaka M Tajima S Kouchi H Protein profile of symbiotic bacteria *Mesorhizobium loti* MAFF303099 in mid-growth phase Biosci Biotechnol Biochem 2003 67 2668 2673 10.1271/bbb.67.2668 14730152

[B10] Hempel J Zehner S Gottfert M Patschkowski T Analysis of the secretome of the soybean symbiont *Bradyrhizobium japonicum* J Biotechnol 2009 140 51 58 10.1016/j.jbiotec.2008.11.002 19095018

[B11] Sarma AD Emerich DW A comparative proteomic evaluation of culture grown vs nodule isolated *Bradyrhizobium japonicum* Proteomics 2006 6 3008 3028 10.1002/pmic.200500783 16688787

[B12] Nomura M Arunothayanan H Dao TV Le HTP Kaneko T Sato S Tabata S Tajima S Differential protein profiles of *Bradyrhizobium japonicum* USDA110 bacteroid during soybean nodule development Soil Sci Plant Nutr 2010 56 579 590 10.1111/j.1747-0765.2010.00500.x

[B13] Sarma AD Emerich DW Global protein expression pattern of *Bradyrhizobium japonicum* bacteroids: a prelude to functional proteomics Proteomics 2005 5 4170 4184 10.1002/pmic.200401296 16254929

[B14] Delmotte N Ahrens CH Knief C Qeli E Koch M Fischer HM Vorholt JA Hennecke H Pessi G An integrated proteomics and transcriptomics reference data set provides new insights into the *Bradyrhizobium japonicum* bacteroid metabolism in soybean root nodules Proteomics 2010 10 1391 1400 10.1002/pmic.200900710 20104621

[B15] Chen H Teplitski M Robinson JB Rolfe BG Bauer WD Proteomic analysis of wild-type *Sinorhizobium meliloti* responses to N-acyl homoserine lactone quorum-sensing signals and the transition to stationary phase J Bacteriol 2003 185 5029 5036 10.1128/JB.185.17.5029-5036.2003 12923075 PMC180974

[B16] Torres-Quesada O Oruezabal RI Peregrina A Jofre E Lloret J Rivilla R Toro N Jimenez-Zurdo JI The *Sinorhizobium meliloti* RNA chaperone Hfq influences central carbon metabolism and the symbiotic interaction with alfalfa BMC Microbiol 2010 10 71 90 10.1186/1471-2180-10-71 20205931 PMC2848018

[B17] Djordjevic MA *Sinorhizobium meliloti* metabolism in the root nodule: a proteomic perspective Proteomics 2004 4 1859 1872 10.1002/pmic.200300802 15221743

[B18] Barra-Bily L Fontenelle C Jan G Flechard M Trautwetter A Pandey SP Walker GC Blanco C Proteomic alterations explain phenotypic changes in *Sinorhizobium meliloti* lacking the RNA chaperone Hfq J Bacteriol 2010 192 1719 1729 10.1128/JB.01429-09 20081032 PMC2832530

[B19] Gao MS Chen HC Eberhard A Gronquist MR Robinson JB Connolly M Teplitski M Rolfe BG Bauer WD Effects of AiiA-mediated quorum quenching in *Sinorhizobium meliloti* on quorum-sensing signals, proteome patterns, and symbiotic interactions Mol Plant Microbe Interact 2007 20 843 856 10.1094/MPMI-20-7-0843 17601171

[B20] Gao MS Chen HC Eberhard A Gronquist MR Robinson JB Rolfe BG Bauer WD sinI- and expR-dependent quorum sensing in *Sinorhizobium meliloti* J Bacteriol 2005 187 7931 7944 10.1128/JB.187.23.7931-7944.2005 16291666 PMC1291280

[B21] Larrainzar E Wienkoop S Weckwerth W Ladrera R Arrese-Igor C Gonzalez EM *Medicago truncatula* root nodule proteome analysis reveals differential plant and bacteroid responses to drought stress Plant Physiol 2007 144 1495 1507 10.1104/pp.107.101618 17545507 PMC1914115

[B22] Knief C Delmotte N Vorholt JA Bacterial adaptation to life in association with plants - A proteomic perspective from culture to in situ conditions Proteomics 2011 11 3086 3105 10.1002/pmic.201000818 21548095

[B23] Koch M Delmotte N Rehrauer H Vorholt JA Pessi G Hennecke H Rhizobial adaptation to hosts, a new facet in the legume root-nodule symbiosis Mol Plant Microbe Interact 2010 23 784 790 10.1094/MPMI-23-6-0784 20459317

[B24] Motokawa M Kobayashi H Ishizuka N Minakuchi H Nakanishi K Jinnai H Hosoya K Ikegami T Tanaka N Monolithic silica columns with various skeleton sizes and through-pore sizes for capillary liquid chromatography J Chromatogr A 2002 961 53 63 10.1016/S0021-9673(02)00133-4 12186391

[B25] Iwasaki M Miwa S Ikegami T Tomita M Tanaka N Ishihama Y One-dimensional capillary liquid chromatographic separation coupled with tandem mass spectrometry unveils the *Escherichia coli* proteome on a microarray scale Anal Chem 2010 82 2616 2620 10.1021/ac100343q 20222674

[B26] Aoki W Ueda T Tatsukami Y Kitahara N Morisaka H Kuroda K Ueda M Time-course proteomic profile of *Candida albicans* during adaptation to a fetal serum Pathog Dis 2013 67 67 75 10.1111/2049-632X.12003 23620121

[B27] Morisaka H Matsui K Tatsukami Y Kuroda K Miyake H Tamaru Y Ueda M Profile of native cellulosomal proteins of *Clostridium cellulovorans* adapted to various carbon sources AMB Express 2012 2 37 41 10.1186/2191-0855-2-37 22839966 PMC3444338

[B28] Masson-Boivin C Giraud E Perret X Batut J Establishing nitrogen-fixing symbiosis with legumes: how many rhizobium recipes? Trends Microbiol 2009 17 458 466 10.1016/j.tim.2009.07.004 19766492

[B29] Shingler V Signal sensory systems that impact Sigma 54-dependent transcription FEMS Microbiol Rev 2011 35 425 440 10.1111/j.1574-6976.2010.00255.x 21054445

[B30] McGarvey DJ Croteau R Terpenoid metabolism Plant Cell 1995 7 1015 1026 7640522 10.1105/tpc.7.7.1015 PMC160903

[B31] Kouchi H Imaizumi-Anraku H Hayashi M Hakoyama T Nakagawa T Umehara Y Suganuma N Kawaguchi M How many peas in a pod? Legume genes responsible for mutualistic symbioses underground Plant Cell Physiol 2010 51 1381 1397 10.1093/pcp/pcq107 20660226 PMC2938637

[B32] Young KD Bacterial shape Mol Microbiol 2003 49 571 580 12914007 10.1046/j.1365-2958.2003.03607.x

[B33] Smit G Kijne JW Lugtenberg BJ Roles of flagella, lipopolysaccharide, and a Ca^2+^−dependent cell surface protein in attachment of *Rhizobium leguminosarum* biovar viciae to pea root hair tips J Bacteriol 1989 171 569 572 2914856 10.1128/jb.171.1.569-572.1989 PMC209624

[B34] Mattick JS Type IV pili and twitching motility Annu Rev Microbiol 2002 56 289 314 10.1146/annurev.micro.56.012302.160938 12142488

[B35] Beringer JE R factor transfer in *Rhizobium leguminosarum* J Gen Microbiol 1974 84 188 198 10.1099/00221287-84-1-188 4612098

[B36] Kawaguchi M *Lotus japonicus* 'Miyakojima' MG-20: An early-flowering accession suitable for indoor handling J Plant Res 2000 113 507 509 10.1007/PL00013961

[B37] Becard G Fortin JA Early events of vesicular arbuscular mycorrhiza formation on Ri T-DNA transformed roots New Phytolog 1988 108 211 218 10.1111/j.1469-8137.1988.tb03698.x 33874168

