# Supplementary material for: Disclosure of the differences of Mesorhizobium loti under the free-living and symbiotic conditions by comparative proteome analysis without bacteroid isolation
Source: BMC Microbiol. 2013 Jul 31;13:180. doi: 10.1186/1471-2180-13-180 (PMC3750425; doi:10.1186/1471-2180-13-180)
Supplement: Additional file 2 — The Venn diagrams of identified proteins at each measurement (N = 3). The number of identified proteins were shown in bold, and percentages were indicated between brackets. [file 1471-2180-13-180-S2.pptx]

## Slide 1
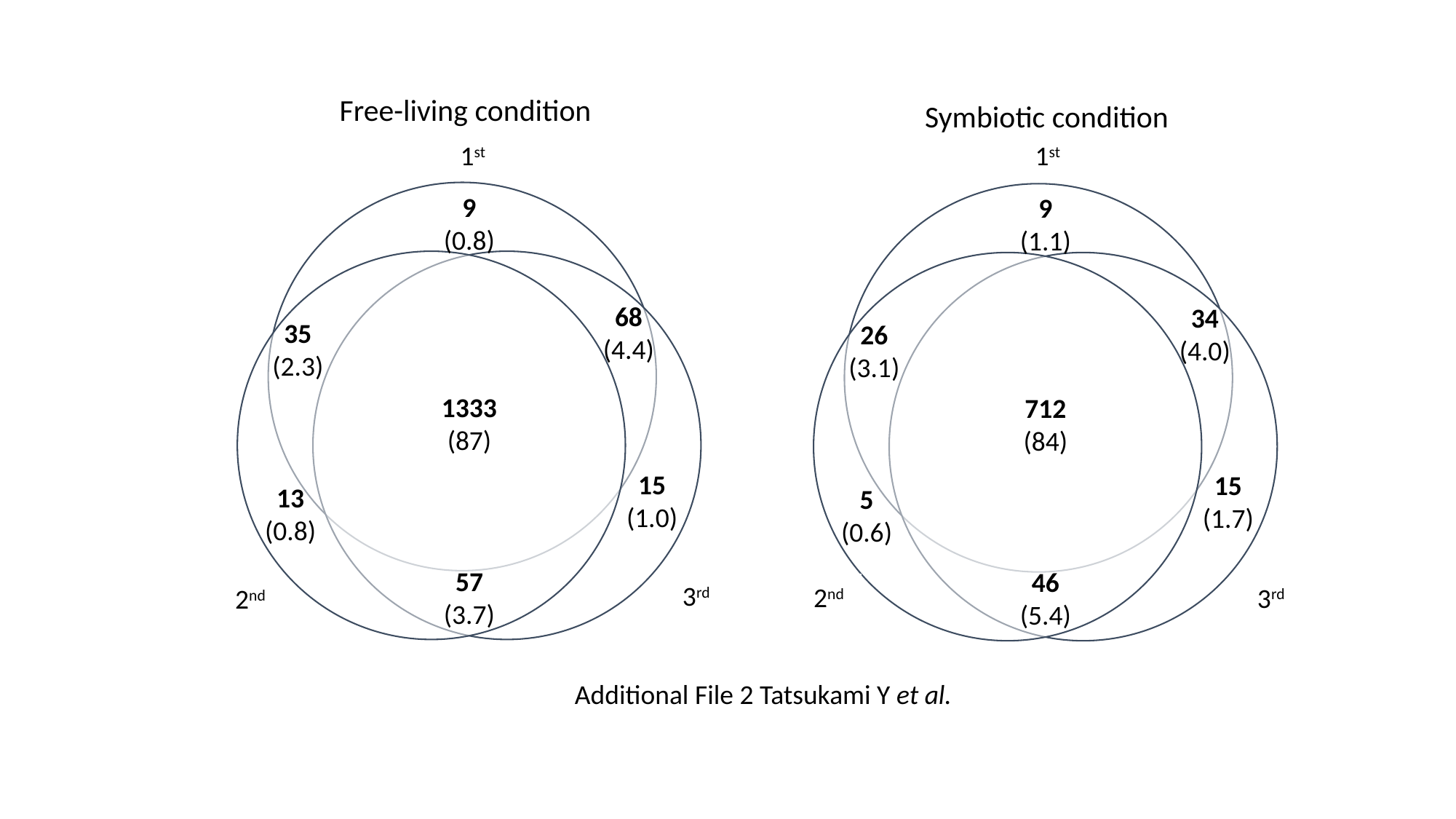

Free-living condition
Symbiotic condition
1st
1st
9
(0.8)
9
(1.1)
68
(4.4)
34
(4.0)
35
(2.3)
26
(3.1)
1333
(87)
712
(84)
15
(1.0)
15
(1.7)
13
(0.8)
5
(0.6)
57
(3.7)
46
(5.4)
3rd
2nd
3rd
2nd
Additional File 2 Tatsukami Y et al.
